# Feasibility and Potential Clinical Ramifications of Using Bacteriophage Therapy for *S. aureus* Necrotizing Fasciitis

**DOI:** 10.3390/jcm14165609

**Published:** 2025-08-08

**Authors:** James B. Doub, Dakarai Dunbar, Sara Jain, Maggie Manchester, Lila Berle, Janvi Madhiwala, Bradley Anderson, Riva Malick, Max Jacobs, Kenneth L. Urish

**Affiliations:** 1 The Doub Laboratory of Translational Bacterial Research, University of Maryland School of Medicine, Baltimore, MD 21201, USA; 2 Division of Clinical Care and Research, Institute of Human Virology, University of Maryland School of Medicine, Baltimore, MD 21201, USA; 3 Department of Orthopedic Surgery, University of Pittsburgh School of Medicine, Pittsburgh, PA 15260, USA

**Keywords:** bacteriophage, necrotizing fasciitis, *Staphylococcus aureus*, enzymes, virulence

## Abstract

**Background:** *Staphylococcus aureus* necrotizing fasciitis is a life-threatening infection requiring aggressive surgical and medical management. Despite these interventions, tremendous morbidity and mortality occur. Thus, novel agents are needed to reduce these negative outcomes. Consequently, the aims of this translational study were to evaluate the feasibility of using bacteriophages and the potential clinical ramifications of using bacteriophages in treatment of *S. aureus* necrotizing fasciitis. **Methods:** Necrotizing fasciitis clinical isolates (n = 6) were tested against different Staphylococcal bacteriophages (n = 4) to assess for activity. After exposure to bacteriophages that had growth inhibition for more than 16 h, the ability of *S. aureus* to change phenotypic expression of numerous enzymes was evaluated, and the ability to reduce bacterial virulence was measured with the *Caenorhabditis elegans* assay. **Results:** Staphylococcal myoviridae bacteriophages were able to lyse most clinical isolates (83%). Interestingly, after exposure to myoviridae bacteriophages, *S. aureus* isolates had no expression of hemolysin, secreted coagulase, or lecthinase, or the ability to ferment mannitol. These same bacteriophages also caused statistically significant decreases in bacterial virulence (*p* < 0.05). Neither findings were observed for bacteriophages of the podoviridae family. **Conclusions:** To use bacteriophages for *S. aureus* necrotizing fasciitis, cocktails of Staphylococcal myoviridae are likely needed to allow for broad host ranges, mitigating the need for in vitro sensitivity testing. Moreover, Staphylococcal myoviridae have the potential to reduce specific enzyme expression and global virulence of residual *S. aureus*. Thus, bacteriophages may aid in reducing necrotizing fasciitis morbidity by not only lysing *S. aureus* but also by reducing *S. aureus* virulence.

## 1. Introduction

Necrotizing fasciitis (NF) is a devastating infection where virulent pathogens instigate profound inflammatory responses leading to tissue necrosis, exposing patients to significant morbidity and mortality [1,2]. Categorization of NF is based on microbial causes, with Type I being polymicrobial and Type II being associated with either *Staphylococcus aureus* or *Streptococcus pyogenes* [2]. Treatment requires prompt surgical debridement in combination with intravenous antibiotics [1,2,3]. Surgical debridement is paramount to remove necrotic tissues, which harbor virulent pathogens. Yet even with these aggressive treatments, many patients need numerous surgical interventions, and amputations are common, especially with *S. aureus* NF [1,2,3]. This occurs because NF *S. aureus* strains secrete numerous enzymes, triggering robust inflammatory responses leading to rapid tissue necrosis [1,2,3]. Consequently, novel approaches are drastically needed to reduce the morbidity and mortality associated with *S. aureus* NF by decreasing microbial burdens and diminishing bacterial virulence.

Some novel approaches, such as hyperbaric oxygen therapy and intravenous immunoglobulin therapy, have been theorized to be advantageous by enhancing the host’s innate immune response [4,5,6]. However, these interventions have limited data to support their use as adjuvant agents in *S. aureus* NF [4,5,6]. Another novel therapeutic that has yet to be evaluated for *S. aureus* NF is bacteriophage therapy. This therapy has the potential to not only aid in eradicating *S. aureus* through bacterial lysis but also reduce bacterial virulence [7]. Yet the use of bacteriophage therapy is complicated in part by the narrow spectrum of activity, and a paucity of translational research hinders the ability of clinicians to determine how bacteriophages will enhance NF treatments [8]. Consequently, the aim of this pilot translational study was to evaluate the ability of Staphylococcal bacteriophages to lyse *S. aureus* NF clinical isolates and reduce *S. aureus* virulence and enzyme expression to thereby devise ways to use bacteriophages for *S. aureus* NF.

## 2. Materials and Methods

### 2.1. S. aureus NF Clinical Isolates

This study was approved by the University of Maryland internal review board (HP-00109342) and the Institutional Biosafety Committee (IBC-00007724). Cases of necrotizing fasciitis from 1 January 2022–1 January 2024 were identified by use of the International Statistical Classification of Diseases and Related Health Problems-10 code M72.6. Patients who had type I necrotizing fasciitis were excluded. Patients who had type II NF (either *S. pyogenes* or *S. aureus*) were included. Manual review of medical records was performed to ensure the NF diagnosis. Cases that did not undergo surgical intervention to confirm NF were excluded. Preserved *S. aureus* NF clinical isolates from the January 2022–January 2024 cases that had been frozen at −80 °C were unfrozen and swabbed onto tryptic soy agar plates and incubated at 37 °C. The isolates that grew were utilized for translational studies.

### 2.2. Bacteriophage Activity Againts S. aureus NF Clinical Isolates

Evaluation of bacteriophage activity against the *S. aureus* NF clinical isolates was conducted by growing the bacterial isolates overnight in tryptic soy broth. Concentrations of this bacterial growth were then diluted to 1 × 10^6^ colony-forming units per mL (CFU/mL). Plaque-forming assays and growth inhibition assays were performed with 4 different bacteriophages (K, Remus, 44A, and 68). 44A and 68 are podoviridae, and Remus and K are myoviridae Staphylococcal bacteriophages. The ability to form plaques was recorded, and then the ability to inhibit bacterial growth for more than 16 h was also evaluated. This was conducted by adding 1 × 10^8^ PFU/mL of each individual bacteriophage to 1 × 10^6^ CFU/mL of each individual clinical isolate and monitoring changes in optical density (600 nm) over time with SpectraMax iD5 (Molecular devices, Sunnyvale, CA, USA). Combinations of bacteriophages were also evaluated for enhanced activity.

### 2.3. Phenotypic Enzyme Variation Before and After Bacteriophage Therapy

To evaluate the phenotypic enzyme variation after exposure to different bacteriophages, individual *S. aureus* strains were first grown overnight and then swabbed onto DNase agar, mannitol fermentation agar, Baird-Parker agar, and blood agar plates. Furthermore, slide coagulase test and test tube coagulation test were conducted. The same strains were then exposed to bacteriophages that could form plaques and inhibit growth for more than 16 h. Bacteria that overgrew the bacteriophages after periods of growth inhibition (˃16 h) were then swabbed onto the same agar plates as indicated above, and the slide and test tube coagulase tests were conducted. When there were changes to phenotypic expression after exposure to bacteriophages, these isolates were then grown in the absence of bacteriophages on tryptic soy agar and retested using the same agar plates and coagulase tests. The experiments were conducted in triplicate and repeated.

### 2.4. Changes in Global Virulence Seen with Caenorhabditis Elegans Assay

Changes in bacterial virulence were assessed by use of the *C. elegans* assay, as has been discussed by others [9,10]. Again, bacterial isolates were grown overnight and then diluted to concentrations of 1 × 10^6^ CFU/mL. These bacteria were then placed on plates where ten L4 *C. elegans* were present and observed for survival over 7 days. This was repeated for the bacteria that overgrew the bacteriophages after periods of growth inhibition (˃16 h). Pairwise comparisons of individual *S. aureus* strain survival after exposure to podoviridae or myoviridae were compared to the same strains before exposure to bacteriophages. Statistical evaluation was conducted using GraphPad Prism 10 (Graphpad software Inc., Boston, MA, USA), in which Chi-squared testing and log-rank test were utilized for comparison. A *p*-value < 0.05 was considered significant. The experiments were conducted in triplicate and repeated.

## 3. Results

From 1 January 2022–1 January 2024, there were 72 patients who had type II NF cases at the University of Maryland Medical Center who underwent surgical debridement and antibiotic therapy. From these cases, 40 were caused by *S. aureus* cases and 32 were caused by *S. pyogenes*. From this period, six *S. aureus* NF clinical isolates had been preserved, allowing for evaluation with the translational studies described above.

**Figure 1:**
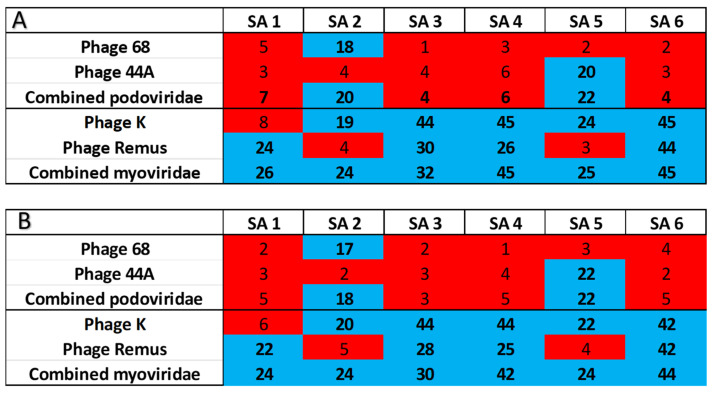
Results of bacteriophage-induced growth inhibition assays for each *S. aureus* clinical isolate (SA1-6) with individual bacteriophages and combined bacteriophages of the same family. Growth inhibition times are listed, where growth inhibition above 16 h is represented by blue rectangles and red rectangles represent times less than 10 h: (**A**) Growth inhibition times with initial exposure of *S. aureus* isolates to the different bacteriophages. (**B**) After *S. aureus* strains were exposed to bacteriophages and then serially passed on tryptic soy agar, similar growth inhibition patterns were observed.

All six strains had at least one bacteriophage (K, Remus, 44A, or 68) that formed plaques and could inhibit growth for more than 16 h (Figure 1). However, only the bacteriophages in the myoviridae family (K, Remus) had activity against 83% of the clinical isolates individually (Table 1). When the myoviridae bacteriophages were combined, they had activity against all the clinical isolates, which did not occur when combining the podoviridae bacteriophages (Table 1).

**Figure 2:**
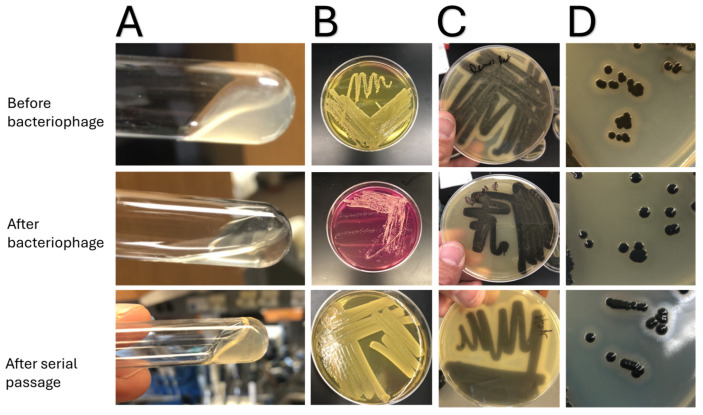
Changes to the phenotypic expression of enzymes before and after exposure to various bacteriophages, as well as after serial passage on tryptic soy agar plates: (**A**) Changes in phenotypic expression of secreted coagulase as seen with the test tube coagulase test. The top picture shows a positive coagulase test before exposure to myoviridae Staphylococcal bacteriophages, and the middle picture shows the same *S. aureus* strain after exposure to Staphylococcal myoviridae in which no coagulase activity is observed. The bottom picture shows phenotypic results after serial passage on agar plates in which reversion back to the wild-type phenotype, which expressed secreted coagulase, was seen. (**B**) Changes in the ability to ferment mannitol. The top picture shows the strains before exposure to the myoviridae Staphylococcal phage in which the bacteria can ferment mannitol. The middle picture shows the same strains after exposure to Staphylococcal myoviridae in which the strains could not ferment mannitol. The bottom picture refers to after serial passage on agar plates in which reversion back to the wild-type phenotype with the ability to ferment mannitol was seen. (**C**,**D**) Changes in lecithinase activity as seen on Baird-Parker agar with addition of egg yolk. All of the *S. aureus* strains before and after exposure to the bacteriophages were capable of reducing tellurite, as seen with black colonies on agar. Before exposure to Staphylococcal myoviridae bacteriophages, lecithinase activity was seen with an opaque zone around colonies (top (**C**,**D**)). Yet after exposure to myoviridae, the *S. aureus* strains did not express this enzyme (middle pictures). However, after serial passage on agar plates, reversion back to the wild-type phenotype in which lectithinase activity was observed (bottom pictures).

Before exposure to bacteriophages, all the *S. aureus* strains could hemolyze red blood cells (beta-hemolysis on blood agar plates), had DNase activity (seen on DNase plates), could ferment mannitol, and had lecithinase activity (seen on Baird-Parker agar) (Figure 2). Moreover, all had positive test tube and slide coagulase tests (Figure 1). After exposure to the podoviridae bacteriophages (44a and/or 68) that had activity against the bacterial isolates, both clinical NF isolates still retained all the enzymatic phenotypic activities assessed. However, when the *S. aureus* strains were exposed to myoviridae bacteriophages (K or Remus) that had activity against the strains, all lost the ability to hemolyze red blood cells, ferment mannitol, lost lecithinase activity, and lost the ability to form coagulase in the test tube test (Figure 1). Yet all the strains retained DNase activity and slide coagulase ability. When these same bacteria were grown on tryptic soy agar devoid of myoviridae bacteriophages, these same strains regained baseline enzymatic abilities after two passages.

**Figure 3:**
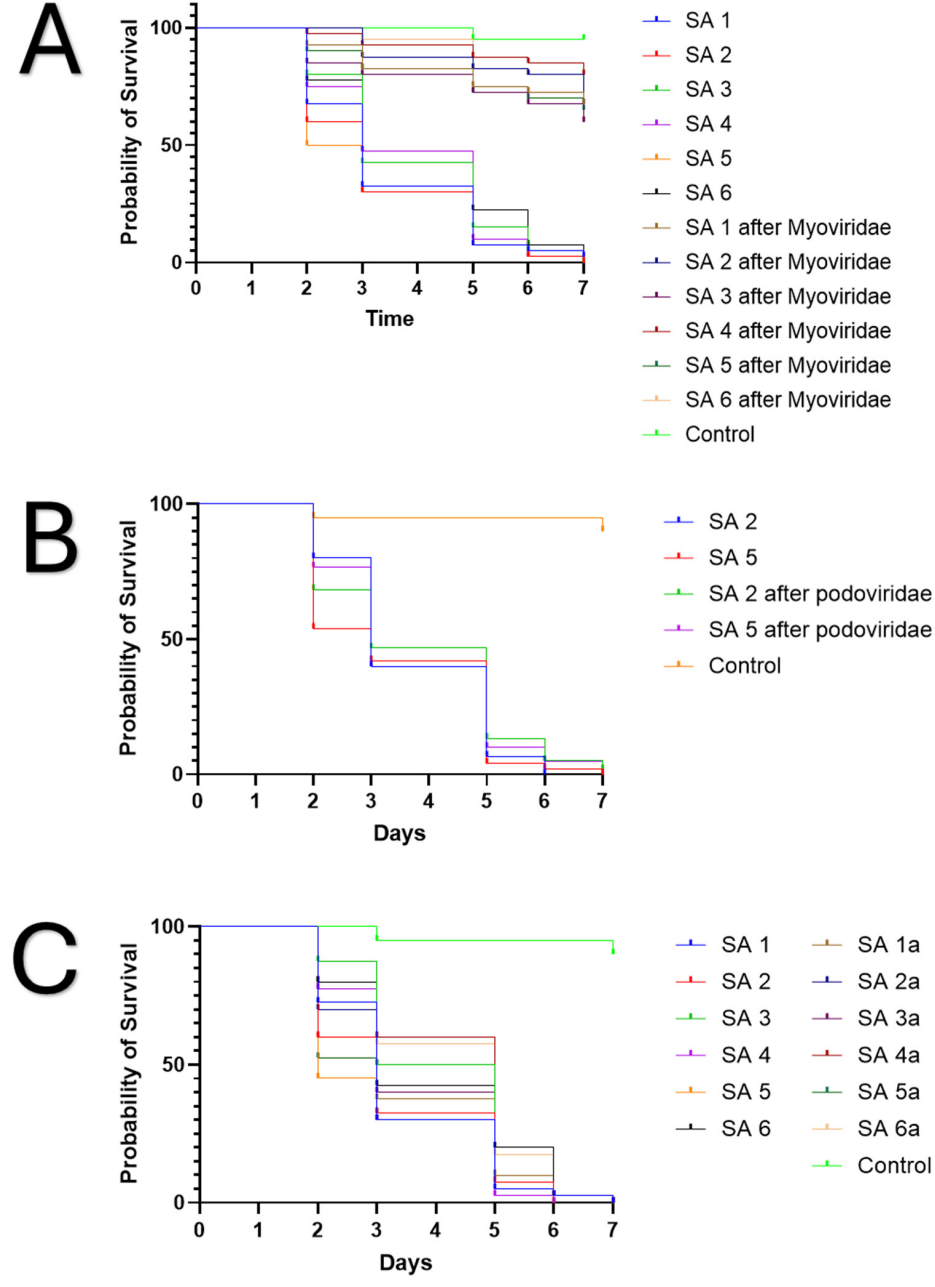
Changes in NF *S. aureus* virulence after exposure to the Podoviridae and Myoviridae Staphylococcal bacteriophages: (**A**) *C. elegans* Kaplan–Meier survival curve, which shows the log-rank test with statistically significant (*p* < 0.0001) decreases in virulence of the *S. aureus* strains before and after exposure to myoviridae as quantified by *C. elegans* mortality after phage predation with myoviridae. (**B**) *C. elegans* survival assay, which shows no statistical difference (*p* = 0.4047) in virulence as quantified by *C. elegans* mortality after phage predation with Podoviridae. (**C**) *C. elegans* survival assay, which shows no statistical difference (*p* = 0.1106) in virulence after isolates were grown on agar devoid of myoviridae bacteriophages.

Furthermore, before exposure to bacteriophages, all the strains killed *C. elegans* within six days. Yet after exposure to myoviridae bacteriophages, which had activity against the individual bacteria, all the strains had a statistically significant decrease in the ability to kill the worms over 7 days (Figure 3A). This was not seen after exposure to podoviridae bacteriophages, in which there was no statistical difference in viability of worms after 7 days (Figure 3B). Moreover, after passage of the strains on tryptic soy agar devoid of bacteriophages, all of them regained their ability to kill *C. elegans*, similar to that seen before exposure to bacteriophages after two passages (Figure 3C). Pairwise statistical calculations can be seen in Supplementary Table S1.

## 4. Discussion

At this nascent stage, bacteriophage therapy is a novel therapeutic that has typically been relegated to aid in treating chronic recalcitrant infections, given that the current treatment paradigm requires a clinical isolate to ensure a bacteriophage therapeutic’s activity in vitro before clinical use [8,11]. This stems from the theoretical narrow spectrum of activity of some bacteriophages, which hinders the ability to use this therapeutic in acute infections, as there is not ample time to conduct this analysis. Yet some bacteriophages are promiscuous with broad host ranges. The ability to have broader host ranges is the result of bacteriophages using binding sites on attachment receptors that are highly conserved amongst a wide variety of bacterial strains [12,13]. As a result, creating therapeutics that utilize these highly conserved binding sites allows the potential to use bacteriophages empirically in acute infections. Thus, understanding bacteriophage attachment receptors is vital to utilizing these agents in acute infections.

Staphylococcal bacteriophages utilize wall teichoic acid (WTA) for attachment, but the two main clinically relevant families of Staphylococcal bacteriophages, podoviridae and myoviridae, bind at different locations on WTA [13]. Staphylococcal podoviridae bacteriophages bind to the distal end of WTA that can have widely different affinities based on the WTA glycosylation patterns [12,13]. On the other hand, Staphylococcal myoviridae bacteriophages bind to the proximal end of WTA that are less impacted by WTA glycosylation [12,13]. Consequently, Staphylococcal myoviridae can have broad host ranges amongst *S. aureus* strains (Table 1) and as seen by others [14,15]. This is important for creating Staphylococcal bacteriophage NF therapeutics, given that there is not ample time to determine in vitro activity. Rather, in order to use bacteriophages for *S. aureus* NF, use of myoviridae bacteriophage cocktails will offer a broad host range and robust lytic activity to numerous clinical strains of *S. aureus*. This will mitigate the need to ensure in vitro activity initially and allow for empiric use of bacteriophage therapy for a large percentage of *S. aureus* NF clinical isolates.

While it is assumed that bacteriophages will lyse all bacteria present, in nature, bacteriophages have evolved to live in predator-prey dynamics with bacteria, and thus the rise and fall of phage populations follow the rise and fall of bacterial populations. Bacteriophages will eradicate a large portion of bacteria, but do not fully eradicate entire bacterial populations. This is commonly seen in vitro after bacteriophage bacterial growth inhibition assays, where bacteria overgrow bacteriophages after a temporal delay [11]. Bacteria evade bacteriophage predation via several mechanisms, but the most common way is by modifying bacteriophage attachment receptors, thereby not allowing for phage attachment [16,17]. For *S. aureus* this is associated with modifications of WTA, which is a major virulence factor [18,19]. Therefore, there might be an evolutionary trade-off in that with WTA modification, there are associated phenotypic changes that can be utilized clinically. In *S. aureus* NF, a reduction in enzyme expression with respect to degradation of lipids and red blood cells, and formation of clots may translate into reduced inflammation and tissue necrosis, thereby reducing morbidity and mortality.

In this study we utilized this principle and evaluated the bacterial overgrowth that occurred after more than 16 h of bacteriophage-induced bacterial growth inhibition. Here we show that all the *S. aureus* NF clinical isolates that evaded Staphylococcal myoviridae bacteriophage predation had less enzymatic activity with respect to hemolysis, lecithinase, secreted coagulase activity, and ability to ferment mannitol (Figure 2). The reduction in enzymatic expression was not universal with respect to the different families of bacteriophages. For podoviridae, no changes in enzyme expression were seen. This likely occurred because podoviridae Staphylococcal bacteriophages bind to distal ends of WTA, and thus post-translational modifications to these regions with respect to glycosylation can easily alter bacteriophage affinity and activity but have little impact on enzyme expression [12,13]. For bacteria to evade Staphylococcal myoviridae bacteriophages, WTA alterations are likely much more pronounced, given that the phage attachment regions are on more proximal portions of WTA and thus, more radical modifications are needed, which drastically change bacterial phenotypic expression of enzymes. Further reinforcing this are other studies implicating WTA in translocation of *S. aureus* enzymes across the cell wall [18]. These findings are important to realize, given that bacteriophages can not only lyse bacterial cells but also reduce specific *S. aureus* enzyme activity, allowing for an NF therapeutic with dual mechanisms of activity.

Interestingly all bacteria, regardless of exposure to myoviridae or podoviridae, still retained DNase activity and slide coagulase activity. This likely occurred because DNAse activity is used by *S. aureus* to degrade extracellular DNA, thereby allowing for escape and spread to other environments, allowing means to escape from phage predation. As for the retention of slide coagulase activity, this likely demonstrates that despite modifications in WTA, *S. aureus* still retains the presence of membrane-bound coagulase, also known as clumping factor. This suggests that global membrane alterations likely do not occur when *S. aureus* evades myoviridae bacteriophage predation, but rather only through subtle changes to WTA. However, this will need to be clarified by use of nuclear magnetic resonance or mass spectrometry to correlate WTA changes with phenotypic changes seen here.

Reducing enzyme expression is important for NF, but the ability to reduce inflammatory processes that are associated with tissue necrosis is also paramount. Here we assessed this by evaluating changes in global bacterial virulence as seen with viability of *C. elegans* to the *S. aureus* strains before and after exposure to bacteriophages. After evasion of myoviridae bacteriophages, *S. aureus* became statistically less virulent compared to the same strain before bacteriophage exposure (Figure 3 and Supplementary Table S1). This did not occur with podoviridae bacteriophages. We hypothesize this likely occurred given the WTA modifications that are needed to evade bacteriophage predation. Yet this was a simplistic approach to assess global virulence, and follow-up studies are needed to assess specific alterations in immune responses that are associated with these findings in other models, such as wax moths or zebra fish [20,21]. Nonetheless, this further demonstrates that Staphylococcal myoviridae have immense promise as *S. aureus* NF therapeutics.

Overall, this was a pilot study that had important clinical findings, but it had several limitations. For one, this study only evaluated gross phenotypic changes. Follow-up studies with nuclear magnetic resonance or mass spectrometry are needed to clarify the mechanism associated with the changes seen here. However, the ability of the bacteria to revert back to virulent states after removing bacteriophage pressures suggests an inducible modification that supports our WTA hypothesis stated above. Secondly, this study only evaluated six NF *S. aureus* strains, and larger studies are needed to confirm the findings seen here and devise cocktails with ample activity against regionally diverse NF clinical isolates. However, this may require prospectively collecting NF *S. aureus* strains, given the potential lack of preserved *S. aureus* NF clinical isolates. Lastly, the clinical strains used did not undergo genomic evaluation to ensure that the same strains were present before and after bacteriophage predation. Yet the similar growth inhibition times after serial passage strongly suggest that these were the same bacterial strains (Supplementary Figure S1).

## 5. Conclusions

In conclusion, *S. aureus* NF is a life-threatening infection that drastically needs novel therapeutics to reduce morbidity and mortality. To use bacteriophage therapy for *S. aureus* NF cocktails of Staphylococcal myoviridae are likely needed to allow for broad host ranges, mitigating the need for in vitro sensitivity testing. Moreover, Staphylococcal myoviridae have the ability to not only lyse *S. aureus* but also reduce specific enzyme expression and global virulence of residual *S. aureus*. Therefore, as outlined here, the use of Staphylococcal myoviridae cocktails could potentially be a powerful adjuvant treatment for *S. aureus* NF. Yet follow-up studies are needed to clarify the mechanism associated with the phenotypic changes seen here and to conduct early clinical studies evaluating safety of myoviridae bacteriophages in *S. aureus* NF.

## Figures and Tables

**Table 1:** Isolate coverage as seen with plaque assays and growth inhibition greater than 16 h.

	Podoviridae (P68, 44A)	Myoviridae (K, Remus)
Combined coverage	2/6 (33.3%)	6/6 (100%)
Single phage	1/6 (16.7%) ^1^	5/6 (83.3%) ^2^

^1^ P68 and 44A bacteriophages were only able to have adequate activity against one isolate. ^2^ K and Remus bacteriophages had adequate activity against five of the six isolates.

## Data Availability

The data generated and analyzed during the current study is available upon reasonable request to the corresponding author.

## Supplementary Materials

The following supporting information can be downloaded at: https://www.mdpi.com/article/10.3390/jcm14165609/s1, Figure S1: Bacteriophage induced growth inhibtion curves; Table S1: Pairwise statistical comparisons of *S. aureus* clinical isolates virulence as seen with *C. elegans* survival before and after exposure to various bacteriophages.
