# Supplementary material for: Feasibility and Potential Clinical Ramifications of Using Bacteriophage Therapy for S. aureus Necrotizing Fasciitis
Source: J Clin Med. 2025 Aug 8;14(16):5609. doi: 10.3390/jcm14165609 (PMC12386667; doi:10.3390/jcm14165609)
Supplement: Supplementary file 1 [file jcm-14-05609-s001.zip › jcm-3754813-supplementary.pdf]

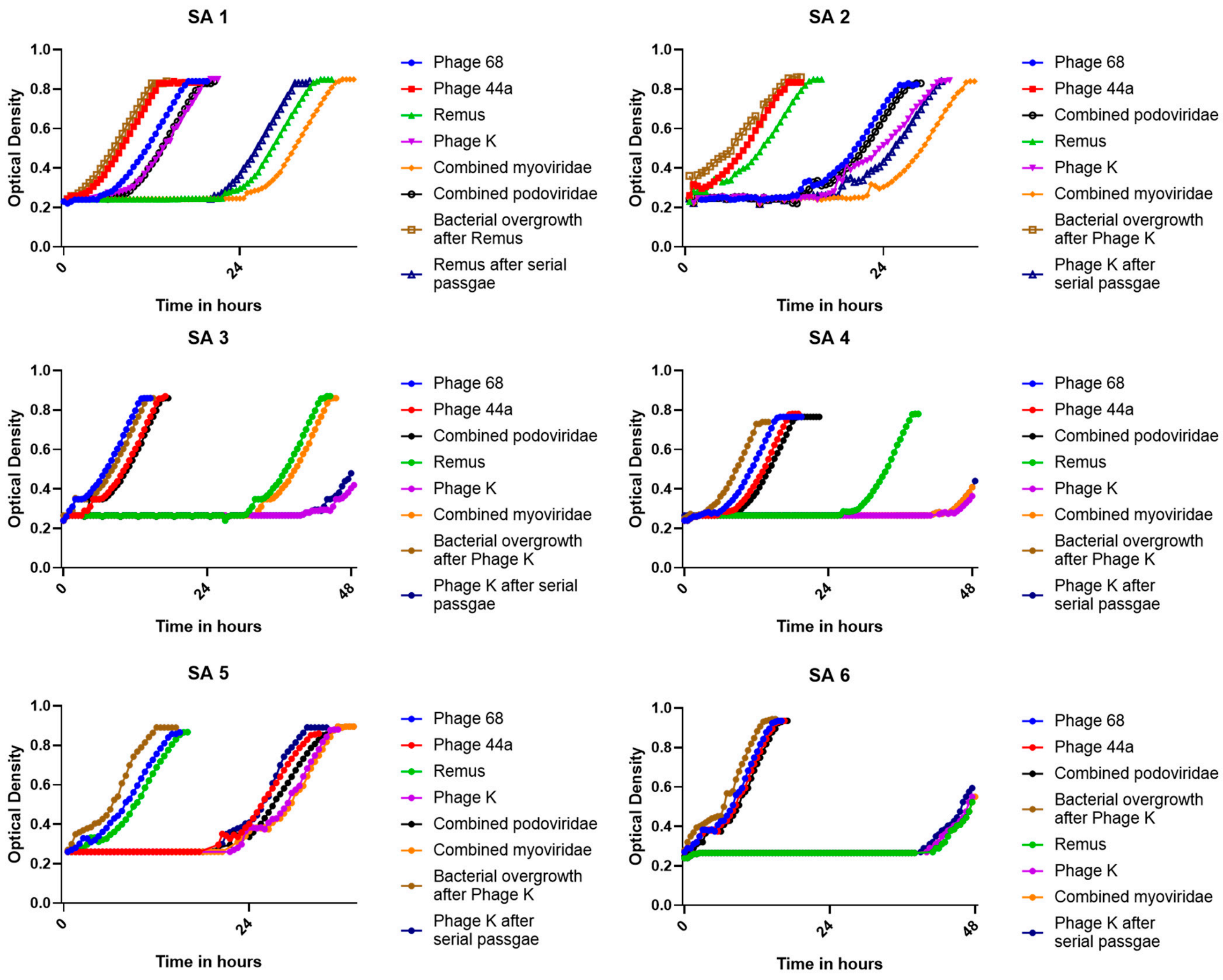

Supplementary Figure S1: Bacteriophage induced growth inhibtion curves. SA 1-6 refers to the different six *Staphylococcus aureus* necrotizing fasciitis clinical isolates. Phages 68 and 44A are podoviridae and Phages K and Remus are myoviridae. Bacterial overgrowth refers to bacteria that grew after exposure to the specific myoviridae bacteriophages indicated and then exposed to the same bacteriophage to demonstrate that the bacteriophage had limited activity. After serial passage refers to the bacteria grown serially on tryptic soy agar and then exposed to the same bacteriophage in which similar growth curves can be seen for all six S. aureus clinical isolates.

Supplementary Table S1: Pairwise statistical comparisons of *S. aureus* clinical isolates virulence as seen with *C. elegans* survival before and after exposure to various bacteriophages

	**P Value**	**95% Confidence intervals**
***S. aureus* isolates virulence before compared to after myoviridae exposure**		
SA 1	P < 0.0001	41.7789% to 70.5005%
SA 2	P < 0.0001	35.6124% to 67.3892%
SA 3	P < 0.0001	40.0463% to 71.7536%
SA 4	P < 0.0001	41.1530% to 71.0791%
SA 5	P < 0.0001	36.3230% to 66.6640%
SA 6	P < 0.0001	42.4512% to 72.0060%
***S. aureus* isolates virulence before compared to after podoviridae exposure**		
SA 2	P = 0.4835	-6.2300% to 11.7836%
SA 5	P = 0.5670	-7.7382% to 14.6286%
***S. aureus* isolates virulence before compared to after myoviridae exposure with serial passage on tryptic soy agar**		
SA 1	P = 0.2363	-4.6289% to 16.4159%
SA 2	P = 0.4682	-7.7382% to 14.6286%
SA 3	P = 0.2974	-6.1656% to 15.5401%
SA 4	P = 0.3827	-6.9317% to 12.5041%
SA 5	P = 0.3209	-4.8397% to 12.5016%
SA 6	P = 0.4616	-5.6408% to 11.0544%
